# Polyphenols from green tea inhibit the growth of melanoma cells through inhibition of class I histone deacetylases and induction of DNA damage

**DOI:** 10.18632/genesandcancer.52

**Published:** 2015-01

**Authors:** Ram Prasad, Santosh K. Katiyar

**Affiliations:** ^1^ Department of Dermatology, University of Alabama at Birmingham, Birmingham, AL, USA; ^2^ Environmental Health Sciences, University of Alabama at Birmingham, Birmingham, AL, USA; ^3^ Nutrition Obesity Research Center, School of Public Health, University of Alabama at Birmingham, Birmingham, AL, USA; ^4^ Comprehensive Cancer Center, University of Alabama at Birmingham, Birmingham, AL, USA; ^5^ Birmingham Veterans Affairs Medical Center, Birmingham, AL, USA

## Abstract

Melanoma is the leading cause of skin cancer-related deaths. We have examined the effect of green tea polyphenols (GTPs), a natural mixture of epicatechin monomers, on melanoma cancer cell growth and the molecular mechanism underlying these effects using different human melanoma cell lines as an *in vitro* model. Treatment of melanoma cell lines (A375, Hs294t, SK-Mel28 and SK-Mel119) with GTPs significantly inhibited the cell viability as well as colony formation ability of melanoma cells in a dose-dependent manner. These effects of GTPs were associated with a significant inhibition of histone deacetylase (HDAC) activity, reduction in the levels of class I HDAC proteins, enhancement of histone acetyltransferase (HAT) activity and induction of DNA damage, as detected by Comet assay, in melanoma cells. GTPs-induced decrease in the levels of class I HDAC proteins is mediated through proteasomal degradation. Valproic acid, an inhibitor of HDACs, exhibited a similar pattern of reduced viability and induction of death of melanoma cells. Treatment of A375 and Hs294t cells with GTPs resulted in a decrease in the levels of cyclins and cyclin dependent kinases of G1 phase of cell cycle whereas upregulated the levels of tumor suppressor proteins (Cip1/WAF1/p21, p16 and p53).

## INTRODUCTION

Melanoma is the leading cause of death related to skin cancer. The average survival of patients with advanced stage melanoma is less than a year because no therapies are effective once the tumor has spread to vital organs [1]. The statistical analysis from American Cancer Society indicated that in 2012, there were 9,180 melanoma-associated deaths in the U.S. and the number of new cases of invasive melanoma was estimated at 76,250 [2]. Although, efforts have been focused on understanding the mechanism of melanoma progression, but the controlling of melanoma has been unsuccessful and yet a challenging task. In addition to environmental factors, epigenetic alterations play an important role in the melanoma progression by altering the expression levels and functioning of various tumor suppressor genes. Epigenetic alterations such as histone modifications, particularly acetylation and deacetylation, are the major driving force for epigenetic gene regulation, which are regulated by two key enzymes: histone deacetylases (HDACs) and histone acetyltransferases (HAT) [3]. Histone deacetylation is associated with transcriptional repression, including a decrease in the expression level of tumor suppressor genes [4]. Several studies reported consistent overexpression of HDACs in colon, breast, prostate, lung, and other cancers [5-10]. In the human genome, HDACs have been identified and classified into four classes: Class I (HDAC 1, 2, 3 and 8); Class II (HDAC 4, 5, 6, 7, 9 and 10); Class III (SIRT 1, 2, 3, 4, 5, 6 and 7) and Class IV (HDAC 11) [11]. Class I HDACs play an important role in controlling cell cycle regulation, cell differentiation, and tissue development. Therefore, it is considered that inhibition of histone deacetylation may reverse the epigenetic silencing of tumor suppressor genes/proteins that is frequently observed in cancer, and this has led to the development of various HDAC inhibitors for cancer therapy. Vorinostat (SAHA) is the first HDAC inhibitor to be approved by the US Food and Drug Administration for cutaneous T-cell lymphoma [12]. However, Phase I and Phase II studies demonstrate that pan-HDAC inhibitors may also cause numerous side effects such as bone marrow depression, diarrhea, weight loss, taste disturbances, electrolyte changes, fatigue, and cardiac arrhythmias [13]. Thus, the question arises that future drug development should focus on selective targeting of individual HDAC family members, which possess a critical oncogenic function in cancer cells but no adverse side effects. Some natural plant products have been shown to have anti-carcinogenic effects in multiple animal tumor models and the phytochemicals that have anti-carcinogenic activity and have no significant toxicity *in vivo* are being investigated as potentially effective chemotherapeutic agents for the prevention and treatment of cancers. The potential of some of these phytochemicals has been investigated on histone modifications [14-16].

Green tea is consumed as a popular beverage world-wide. It is largely consumed in some Asian countries such as Japan, China, Korea, and parts of India, and a few countries in North Africa and the Middle East [17, 18]. The consumption of green tea is also increasing in the western countries including the United States because of increasingly new investigations on its health benefits and anti-carcinogenic activities in various organs. The characteristic aroma and health benefits of tea are associated with the presence of catechins/epicatechins and their derivatives, which are commonly called “polyphenols“ or green tea polyphenols (GTPs). The major polyphenols present in green tea are: (−)-epicatechin, (−)-epigallocatechin, (−)-epicatechin-3-gallate, and (−)-epigallocatechin-3-gallate (EGCG) [18, 19]. GTPs have been found to alter various molecular targets that are known to affect tumor cell growth and their survival [18, 20]; however, little is known as to whether GTPs target alterations in epigenetic regulators in cancer or target events subsequent to the initiation of carcinogenic process. As, it is well known that overexpression of class I HDACs plays a crucial role in carcinogenesis, we sought to determine the chemotherapeutic effect of GTPs on melanoma cancer cells and whether it is mediated through its effect on HDACs.

To address this issue, we investigated whether GTPs have the ability to suppress the levels of class I HDAC proteins and their activity in human melanoma cells and whether this effect is associated with their effects on cell growth/viability, cell cycle regulatory proteins and reactivation of tumor suppressor proteins using *in vitro* cell culture model. As melanoma is a leading cause of death due to skin diseases, the exploration and development of new and effective agents that are non-toxic in nature and that can target the molecules associated with epigenetic regulators could lead to substantially improved outcomes in patients with this disease. Here, we report that treatment of melanoma cells with GTPs reduces the melanoma cell viability and this effect of GTPs is mediated through, (i) inhibition of the levels of class I HADC proteins, (ii) inhibition of HDAC activity while enhancing HAT activity, (iii) DNA damage and (iv) suppression of cell cycle regulatory proteins of G1 phase. Thus, our studies provide evidence that GTPs have the ability to inhibit the growth of melanoma cells by targeting epigenetic regulators.

## RESULTS

### GTPs inhibit cell growth and induce cytotoxicity in melanoma cells

**Figure 1:**
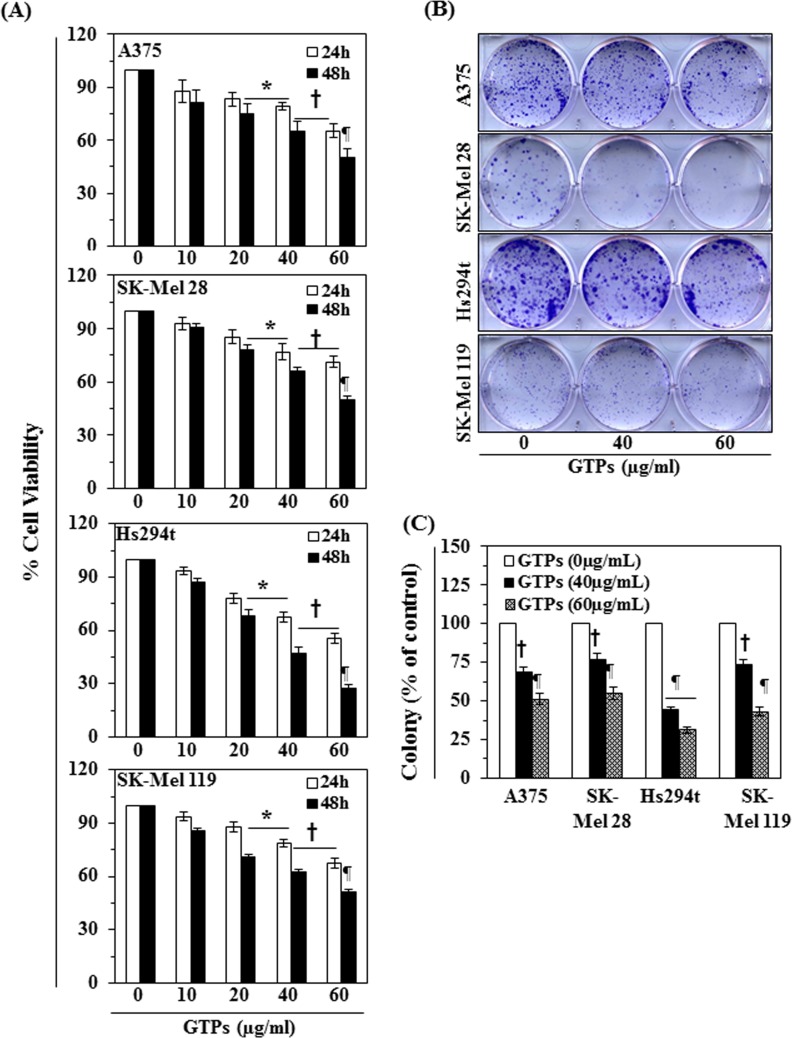
Cytotoxic effect of GTPs on melanoma cells (A) Treatment of human melanoma cells (A375, SK-Mel28, Hs294t, SK-Mel119) with various concentrations of GTPs (0, 10, 20, 40 and 60 μg/ml) inhibits the proliferation or cell viability in a dose- and time-dependent manner. Cell viability was determined using MTT assay as described in the Materials and Methods section, and data are expressed in terms of percent of control group (non-GTPs treated) as the mean ± SD of six replicates. Significant difference versus non-GTPs-treated controls, *P <0.05; ^†^*P*<0.01; ^¶^*P*<0.001. (B) Treatment of melanoma cells with GTPs for 2 weeks inhibits the colony formation ability of cells. Cancer cell colonies are shown in blue-purple. (C) Number of colonies in each treatment group was detected and counted under Olympus microscope and data on colony formation are summarized in terms of percent of control. Significant difference versus control, ^†^*P*<0.01, ^¶^*P*<0.001.

The effect of GTPs on cell viability/proliferation was determined using MTT assay as described previously [10, 21]. Melanoma cell lines, A375, SK-Mel28, Hs294t and SK-Mel 119, were treated with different concentrations of GTPs (0, 10, 20, 40, and 60 μg/ml) for 24 and 48 h. As shown in Fig. 1A, treatment of melanoma cells with GTPs resulted in significant reduction of the cell viability: A375 (12-34% at 24 h and 18-49% at 48 h), SK-Mel 28 (7-29% at 24 h and 9-50% at 48 h), Hs294t (6-45% at 24 h and 13-72% at 48 h), and SK-Mel 119 (6-32% at 24 h and 14-49% at 48 h). The inhibitory effect of GTPs on melanoma cells growth was also verified and tested using colony formation assay, as shown in Fig. 1B. The colonies are shown in purple-dark blue. The individual colonies were counted under microscope and resultant data on colony formation are summarized in Fig. 1C in terms of percent of control (non-GTPs-treated group). As shown in Fig. 1C, treatment of different melanoma cell lines with various concentrations of GTPs significantly inhibited (*P*<0.01, *P*<0.001) the colony formation ability in each melanoma cell line compared with control group (non-GTPs-treated cells). Moreover, the size of the colonies was smaller in GTPs-treated cells compared to control group. These results indicate the cytotoxic action of GTPs in melanoma cells. Importantly, treatment of NHM with GTPs did not result in significant inhibition of cell viability under identical experimental conditions [22].

### Detection of basal level of class I HDAC proteins, HDAC and HAT activity in different melanoma cell lines

**Figure 2:**
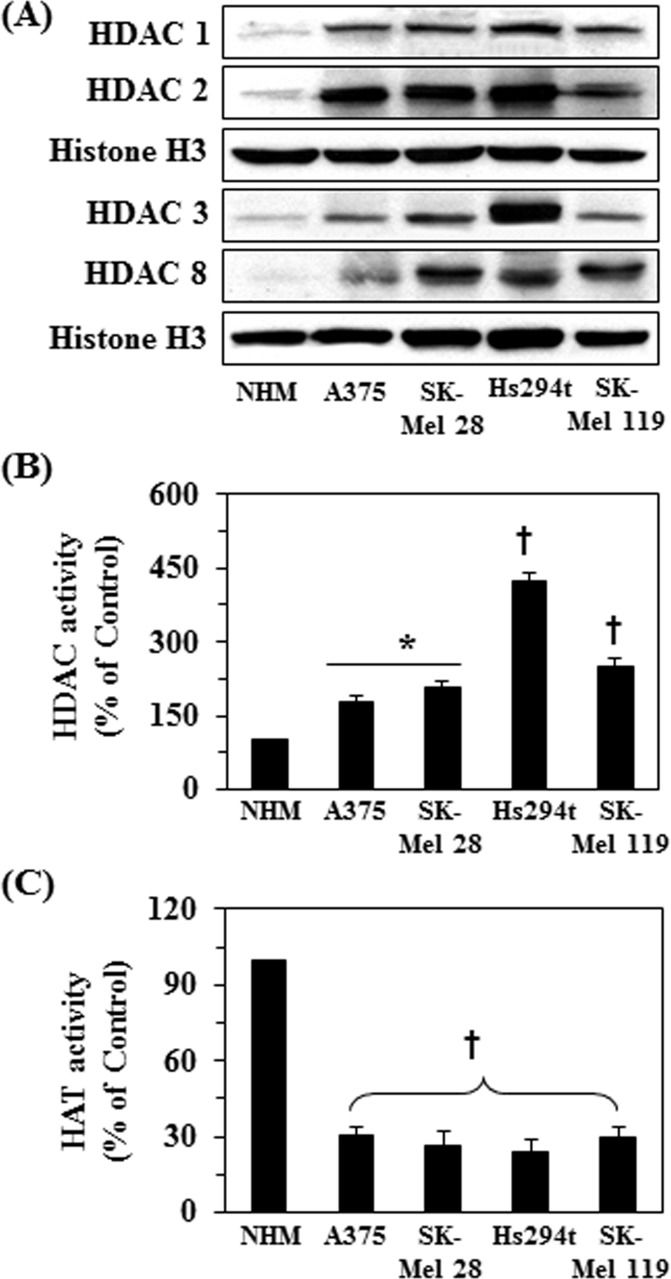
Basal levels of class I HDAC proteins, HDAC and HAT activity in different melanoma cell lines compared to normal human melanocytes (NHM) NHM and melanoma cell lines were cultured, harvested and nuclear fractions were prepared. (A) Expression levels of class I HDACs proteins were analyzed in nuclear fractions using western blot analysis. (B) HDAC activity was measured in cells using HDAC Activity Assay Kit following the manufacturer's instructions, and (C) HAT activity was measured using EpiQuik^TM^ HAT Activity Assay Kit following manufacturer instructions. Data are presented in terms of percent of control cells as mean ± SD), n=3. Significant difference in melanoma cell lines versus NHM, *P <0.01; †*P*<0.001.

To determine the roles of HDACs and HAT activities in melanoma cell growth and the effect of GTPs on them, first we determined the basal expression levels of class I HDACs proteins, HDAC and HAT activities in different melanoma cell lines and compared these data with normal human melanocytes (NHMs). As shown in Figure 2A, western blot analysis revealed that the basal levels of class I HDAC proteins were higher in all the melanoma cell lines compared with NHM. Similarly, the levels of HDAC activity were also significantly higher (*P*<0.05-*P*<0.001) in melanoma cell lines compared with normal human melanocytes, as shown in Figure 2B. Further, the levels of HDAC activity was higher in Hs294t cells compared to other melanoma cell lines studied. Similar to HDAC activity, we also determined the activity of HAT in all the melanoma cell lines and data were compared with NHM. As shown in Figure 2C, HAT activity was significantly lower (more than 3-fold, *P*<0.001) in all the melanoma cell lines compared to NHM. These observations suggest that melanoma cell lines are epigenetically modified and that these modifications in HDAC and HAT activities may have an important role in melanoma progression.

### Effect of GTPs on HDAC and HAT activities in melanoma cells

**Figure 3:**
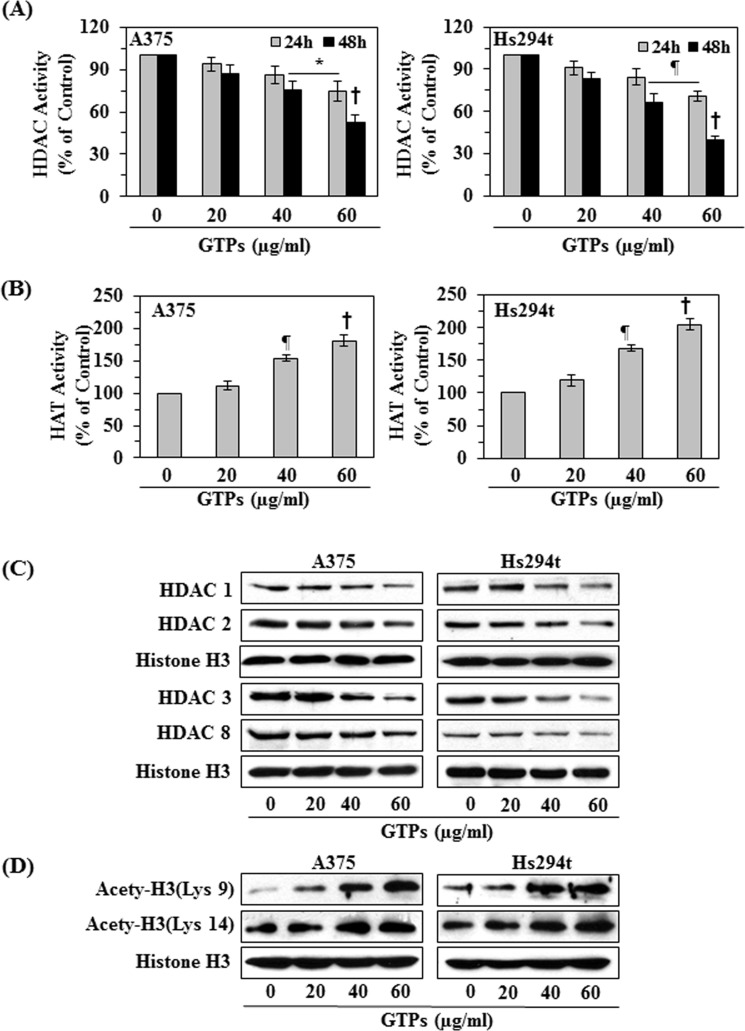
Effect of GTPs on HDAC and HAT activity and the expression levels of class I HDACs proteins in melanoma cell lines A375 and Hs294t melanoma cell lines were treated with various concentrations of GTPs (0, 20, 40, and 60 μg/ml) for 24 or 48 h, and nuclear cell lysates were subjected to activity assays and western blot analysis. (A) The effect of GTPs on HDAC activity was measured using HDAC Activity Assay Kit, following manufacturer's instructions. Significant difference versus non-GTPs-treated control, *P<0.05, ^¶^*P*<0.01, ^†^*P*<0.001. (B) Treatment of melanoma cell lines with GTPs for 48 h enhances HAT activity in cells. Significant difference versus non-GTPs-treated controls, ^¶^*P*<0.01, ^†^*P*<0.001. Data are presented in terms of percent of control as mean ±SD, n=3. (C) Cells were treated with various concentrations of GTPs for 48 h, and cell lysates were subjected to western blot analysis for class I HDAC proteins. GTPs decrease the expression levels of HDAC proteins in melanoma cells. (D) GTPs enhance histone acetylation in melanoma cells when cells were treated for 48 h. Equal loading of protein samples was verified using anti-histone H3 antibody.

To determine the effect of GTPs on HDAC and HAT activities, two representative melanoma cell lines (A375 and Hs294t) were treated with various concentrations of GTPs (0, 20, 40, and 60 μg/ml) for 24 and 48 h. The nuclear fractions were subjected to the analysis of HDAC and HAT activities using their respective analytical kits. As shown in Figure 3A (left panel), GTPs treatment of A375 melanoma cells resulted in significant inhibition of HDAC activity (6-25% at 24 h, and 13-47% at 48 h; *P*<0.05 to *P*<0.001) as compared with vehicle-treated control cells and that this inhibitory effect of GTPs was dose- and time-dependent. Similar effects were also obtained when Hs294t cells were treated with GTPs (Fig. 3A, right panel). The effects of GTPs on HAT activity in A375 and Hs294t cells were determined using the HAT Activity Assay Kit. Treatment of cells with GTPs for 48 h resulted in significantly (*P*<0.01, *P*<0.001) higher levels of HAT activity in both A375 and Hs294t cells as compared to the control cells, which were not treated with GTPs, in a concentration-dependent manner (Figure 3B).

### GTPs treatment decreases protein expression of class I HDACs in melanoma cells

As we observed that melanoma cell lines overexpressed class I HDACs proteins compared to normal human melanocytes (Fig. 2A), we investigated whether GTPs treatment affects the protein expression of class I HDACs in melanoma cell lines. For this purpose A375 and Hs294t cells were treated with GTPs (0, 20, 40 and 60 μg/ml) for 48 h. Cell lysates were subjected to western blot analysis of HDAC proteins. As shown in Figure 3C, western blot analysis revealed that treatment of cells with GTPs resulted in a dose-dependent reduction in the expression levels of the class I HDAC proteins (HDAC1, HDAC2, HDAC3 and HDAC8) in both cell lines as compared with the vehicle-treated control cells.

### GTPs treatment enhances the levels of acetylated histone H3 at lysine 9 and 14 residues and induces DNA damage in melanoma cells

Acetylation of histones is associated with transcriptional activation, and we have observed that the levels of HAT activity were less in melanoma cells compared with normal human melanocytes (Figure 2C), we checked whether GTPs affect the acetylation status of histones in melanoma cancer cell lines. For this purpose, A375 and Hs294t cells were treated with GTPs (0, 20, 40 and 60 μg/ml) for 48 h, then cells were harvested and nuclear lysates were prepared for western blot analysis. Our resultant data revealed that treatment of cells with GTPs resulted in a dose-dependent increase in the levels of the acetylated histone H3 at lysine 9 and lysine 14 compared with the vehicle-treated control cells, as shown in Figure 3D.

**Figure 4:**
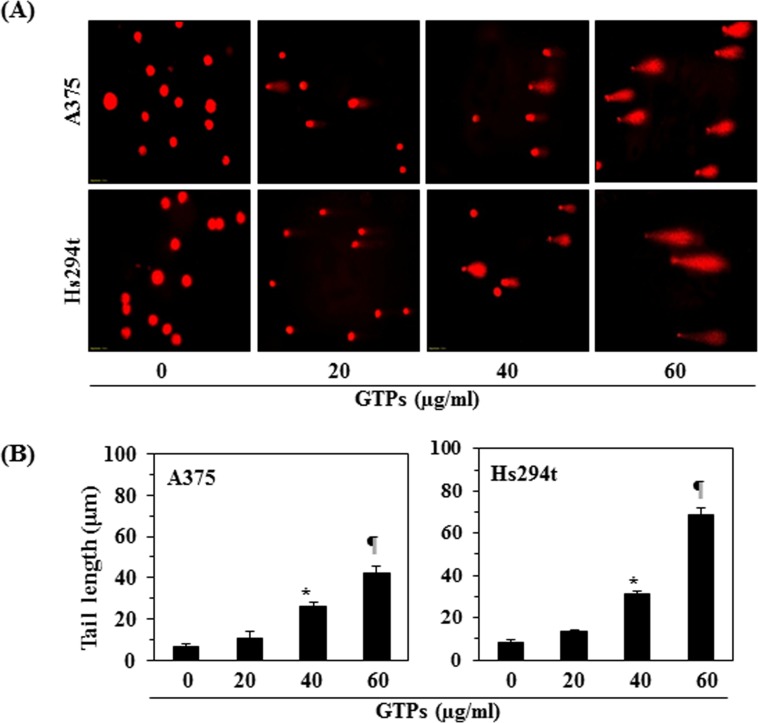
Treatment of melanoma cells with GTPs resulted in DNA damage which was identified by Comet assay (A) A375 and Hs294t cells were treated with different doses of GTPs (0, 20, 40 and 60 μg/ml) for 48 h, then cells were harvested and DNA damage was recognized using Comet assay. (B) The tail length of Comet or damaged DNA was measured in each cell and tail length is expressed in μm as a mean ± SD from at least 6-10 cells in each treatment group. Significant increase in tail length (a marker of DNA damage) versus non-GTPs-treated control group, *P<0.001, ^¶^*P*<0.0001.

As treatment of melanoma cells with GTPs resulted in suppression of class I HDAC proteins expression and restoration of HAT activity (Fig. 3), we determined whether these effects lead to the induction of DNA damage in melanoma cells after treatment with GTPs. To test this effect, A375 and Hs294t cells were treated with GTPs for 48 h, and then harvested cells were subjected to the analysis of DNA damage following Comet assay, as described in Materials and Methods. The data from Comet assay clearly revealed that DNA of melanoma cells was damaged which was recognized in the form of Comet or long tail in cancer cells under microscope, as shown in Figure 4A. The length of Comet or tail in both cell lines was measured in different treatment groups and data are summarized in Figure 4B. Treatment of GTPs resulted in significant increase in the lengths of Comet (*P*<0.001) compared to non-GTPs-treated control cells. These data indicated that GTPs were able to induce DNA damage in melanoma cells and that was resulted in suppression of cell viability of melanoma cells.

### GTPs decrease the expressions of cell cycle regulatory proteins of G1 phase while stimulate reactivation of tumor suppressor proteins in melanoma cells

**Figure 5:**
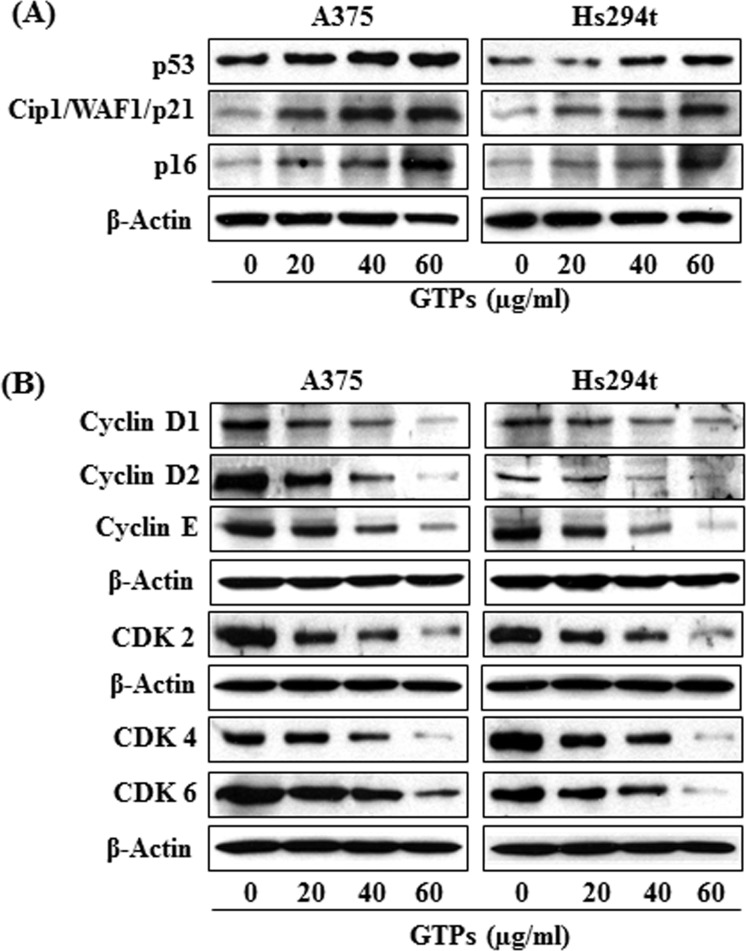
Treatment of A375 and Hs294t melanoma cell lines with GTPs for 48 h resulted in reactivation of tumor suppressor proteins and affects the cell cycle regulatory proteins of G1 phase Melanoma cell lines (A375 and Hs294t) were treated with various concentrations of GTPs for 48 h; then cells were harvested for cell lysates, which were subjected to western blot analysis. (A) Treatment of cells with GTPs enhances or reactivated the expressions of tumor suppressor proteins, such as p53, Cip1/WAF1/p21 and p16. (B) Treatment of cells with GTPs inhibits the levels of cyclins and CDKs associated with the G1 phase of the cell cycle in a dose-dependent manner, as analyzed by western blotting. Equal loading of protein samples was verified using anti-β-actin antibody. Representative blots are shown.

As the treatment of melanoma cells with GTPs resulted in a reduction in Class I HDAC protein expression, DNA damage and cell viability, we next determined whether this effect of GTPs on melanoma cells is associated with deregulation of cell cycle regulatory proteins. For this purpose the effect of GTPs was determined on cell cycle regulatory proteins in A375 and Hs294t cells following treatment of cells with GTPs for 48 h. As shown in Figure 5B, the analysis of cell cycle proteins of G1 phase revealed that treatment of A375 and Hs294t cells with GTPs resulted in inhibition of cyclin D1, cyclin D2 and cyclin E proteins expressions in a dose-dependent manner. Similarly, a marked reduction in the expression of CDK2, CDK4 and CDK6 proteins was observed (Figure 5B). Inhibitory effect of GTPs on cyclins and CDKs of G1 phase in both melanoma cell lines was almost identical. These results suggest that GTPs induce deregulation of G1 phase cell cycle proteins following DNA damage in melanoma cell lines.

HDAC inhibitors induce upregulation of Cip1/WAF1/p21 expression, which is a critical target of p53 and arrests G1 phase of cell cycle [23]. Due to these associations or link, we checked the effect of GTPs on tumor suppressor proteins, Cip1/WAF1/p21, p16 and p53 in melanoma cells. For this purpose, A375 and Hs294t cells were treated with various concentrations of GTPs for 48 h, then cells were harvested and cell lysates prepared. Western blot analysis revealed that treatment of melanoma cells with GTPs resulted in restoration or reactivation of tumor suppressor proteins in a dose-dependent manner, as shown in Figure 5A.

### GTPs-induced reduction of class I HDAC proteins in melanoma cells is mediated through proteasomal degradation of HDACs

**Figure 6:**
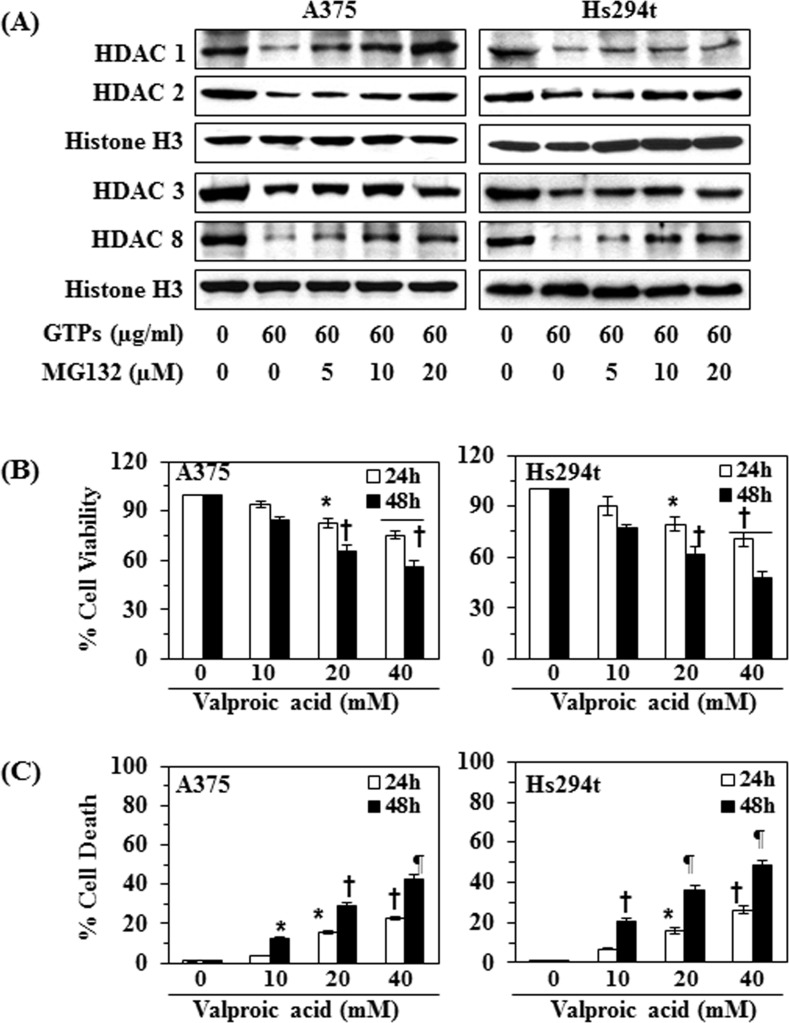
Effect of MG132 (a proteasome inhibitor) on GTPs-induced inhibition of class I HDACs expression in A375 and Hs294t cells (A) Treatment of melanoma cells with MG132 inhibits the effect of GTPs on HDAC protein expressions. A375 and Hs294t cells were treated with GTPs (60 μg/ml) with and without the treatment of MG132 for 48 h, then cells were harvested and nuclear lysates were subjected to western blot analysis. (B) A375 and Hs294t cells were treated with various concentrations of valproic acid (0, 10, 20 and 40 mM) for 24 and 48 h, and cell viability was determined using an MTT assay. Data on cell viability are presented in terms of percent of control group (non-valproic acid-treated) as the mean ±SD of 6 replicates. (C) Treatment of melanoma cells with valproic acid induces cell death. Cell death was determined using a trypan blue exclusion assay. Data are presented in terms of the percent cell death as the mean ± SD from three separate experiments. Significant difference versus non-valproic acid treated controls, *P<0.05, ^†^*P*<0.01, ^¶^*P*<0.001.

To determine whether GTPs reduce the levels of HDAC proteins in melanoma cells through proteasome-mediated degradation, A375 and Hs294t cells were treated with GTPs (60μg/ml) with and without treatment with MG132 (5, 10 and 20 μM conc.), an inhibitor of proteasomal degradation, for 48 h. Cells were harvested and nuclear lysates were prepared for western blot analysis. Western blot analysis revealed that the levels of class I HDAC proteins were higher in the cells treated with GTPs + MG132 as compared with levels in the cells treated with GTPs alone (Figure 6A). These results indicate that proteasome-mediated degradation of HDACs may be a possible mechanism through which GTPs reduce the levels of class I HDACs proteins in both melanoma cell lines.

### Treatment of valproic acid, an inhibitor of HDAC, reduces the cell viability and promotes cell death in melanoma cells

The above results suggested that GTPs-induced inhibition or reduction in cell viability of melanoma cells was associated with the reduction in the expression of HDAC proteins; we further examined the effects of valproic acid, a well known inhibitor of HDAC, on the viability and death of melanoma cells to verify that the action of GTPs is similar to that of HDAC inhibitor. Treatment of A375 and Hs294t cells with various concentrations of valproic acid (0, 10, 20 and 40 mM) for 24 and 48 h resulted in a significant reduction (*P*<0.05, *P*<0.001) in cell viability in a time- and dose-dependent manner, as assessed using an MTT assay (Figure 6B). Treatment of the melanoma cells with valproic acid also resulted in a significant (*P*<0.001) higher levels of cell death as compared with the non-valproic acid-treated control cells (Figure 6C). These observations suggest that the cytotoxic effects of GTPs on the melanoma cells are similar to those of an inhibitor of Class I HDACs.

## DISCUSSION

Overexpression of class I HDACs has been identified in human cancers and therefore HDACs are considered to be the promising targets in oncology and epigenetic therapy [24, 25]. Acetylation and deacetylation are the main histone modifications that have been clinically identified as predictors of cancer progression [24, 26, 27]. In the current study, we have found that the expression levels as well as activity of class I HDACs are elevated in the human melanoma cells lines (A375, Hs294t, SK-Mel28 and SK-Mel119) as compared to the normal human melanocytes. Additionally, the activity of HAT also was lower in these melanoma cells than normal human melanocytes. Some clinical trials with synthetic HDAC inhibitors have demonstrated promising therapeutic activity and therefore HDACs have become prime targets in cancer drug development. The outcome of preclinical studies indicate that inhibitors of HDAC can modulate a wide variety of cellular functions, such as cell cycle progression, angiogenesis and apoptosis, etc. [25]. HDAC inhibitors, such as suberoylanilide hydroxamic acid (SAHA) and trichostatin A (TSA), have been shown to induce apoptosis in neoplastic cells *in vitro* and inhibit tumor growth *in vivo* in animal models [28-31]. GTPs have been shown to have anti-nonmelanoma skin cancer potential in various *in vivo* animal models [17-20]. In the current study, we demonstrate that treatment of various human melanoma cell lines *in vitro* (A375, Hs294t, SK-Mel28 and SK-Mel119) with GTPs significantly inhibits their cell viability but this effect of GTPs was not observed in normal human epidermal melanocytes. GTPs also induce toxicity in melanoma cells which is indicated by their inhibitory effects on colony formation (Fig. 1B). The inhibition of cell proliferation, cell viability or cytotoxicity in melanoma cells by GTPs is associated with the inhibition of HDAC activity and reduction in the levels of class I HDAC proteins in a dose- and time-dependent manner, while elevated HAT activity (Figure 3). Class I HDACs are responsible for deacetylation of the catalytic core for different co-repressor complexes resulting in transcriptional repression. Interestingly, GTPs suppressed the levels of all class I HDAC proteins while enhanced the acetylation of histones which is a protective mechanism and may contributed in reactivation of tumor suppressor proteins. This study also provides evidence that GTPs suppress the levels of HDAC proteins in melanoma cells through their proteasomal degradation. This possibility was suggested by the effects of valproic acid, a known HDAC inhibitor, which also reduced cell viability and induced cell death in melanoma cells (Figure 6). These results indicate that the action of GTPs against melanoma cells is similar to that of valproic acid, which is a well known synthetic inhibitor of HDAC. Enhanced DNA damage was observed in culture by GTPs as detected by Comet assay. It may be due to induction of histone hyperacetylation by GTPs, resulting in a more open chromatin structure, making DNA more susceptible to damage by therapeutic agents like GTPs. Importantly, HDAC inhibitors have been shown to decrease the expression of DNA repair proteins, such as RAD51 [32]. Other investigations also emphasize/support the role of HDACs in genome surveillance, and HDAC inhibitors appear to facilitate cancer cell death by enhancing the DNA damage response and inhibiting DNA repair [33].

**Figure 7:**
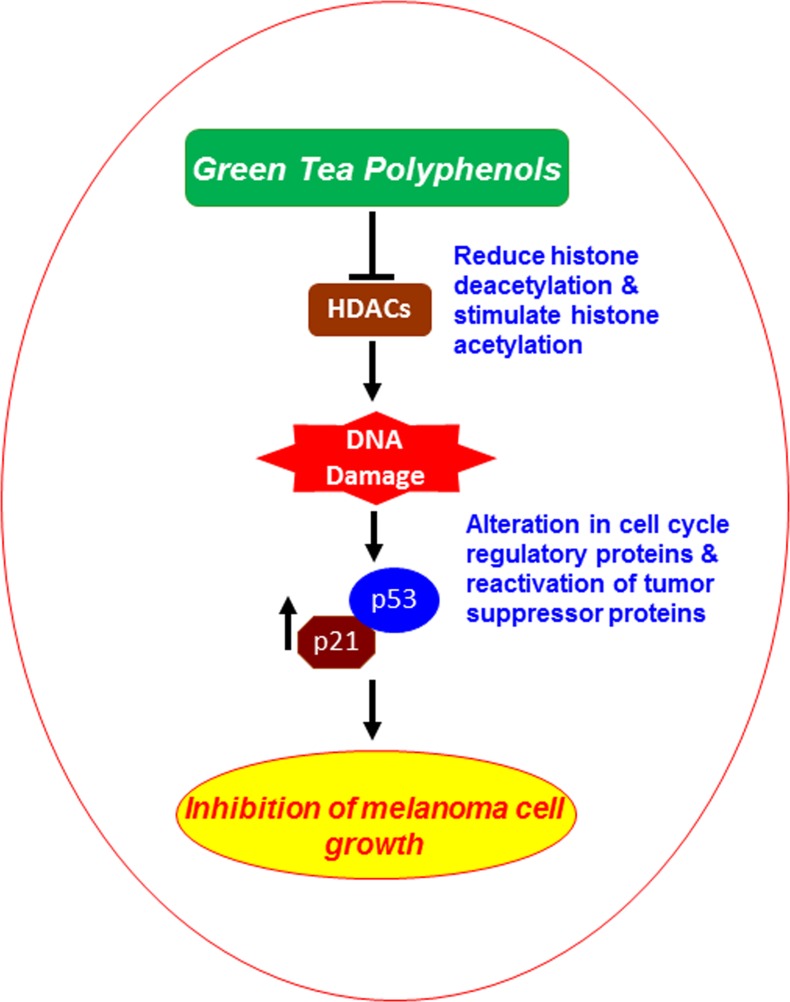
Schematic diagram showing the possible mechanism through which GTPs inhibit melanoma cell growth Inhibition of cell viability/growth by GTPs is mediated via targeting inhibition of class I HDACs proteins, promoting DNA damage and reactivation of tumor suppressor proteins.

It has been identified that cell cycle regulators are frequently mutated or deregulated in most of the human malignancies; therefore, the control of cell cycle progression in cancer cells may be an effective strategy to prevent cancer growth or progression [34-37]. Our study demonstrates that *in vitro* treatment of melanoma cells with GTPs decreases the expressions of cyclins and CDKs (CDK2, CDK4 and CDK6) of G1 phase in both A375 and Hs294t cell lines suggesting that GTPs induce a marked disruption of the uncontrolled cell cycle progression, and that may be a mechanism by which GTPs inhibit the proliferation or suppress the cell viability of melanoma cells. This action of GTPs is associated with the DNA damage and inhibition of HDAC activity in melanoma cells. In summary, our findings are of importance for understanding the anti-melanoma effect of GTPs, related mechanisms and clinical applications of GTPs in human system, as summarized in Figure 7. Further, the new insights into the epigenetic mechanism of action of GTPs may contribute to the chemoprevention or treatment of melanoma and may have important implications for epigenetic therapy. The use of GTPs in combination with other known HDAC inhibitors may be more effective for the treatment of melanoma and needs to be examined and explored in *in vivo* systems.

## MATERIALS AND METHODS

### Green tea polyphenols

The purified mixture of GTPs (Sunphenon 90D, Food grade, purity >90% polyphenols) was obtained from Taiyo International Inc. (Minneapolis, MN), which contains primarily five major epicatechin derivatives, such as (−)-epigallocatechin-3-gallate, (−)-epigallocatechin, (−)-epicatechin gallate and (−)-epicatechin. For the treatment of cells, GTPs were dissolved in small amount of PBS buffer and mix into cell culture medium to acquire desired concentration into subconfluent cells (60-70% confluent). Importantly, we used the mixture of green tea polyphenols (GTPs) in this study with the assumption that all ingredients may act together additively or synergistically and may be better than a single constituent. Moreover, the use of GTPs is more practical as people consume water extract of green tea as a popular beverage.

### Antibodies, chemicals and reagents

The antibodies specific for HDAC1, HDAC2, HDAC3, HDAC8, Cip1/Waf1/p21, p16, CDK2, CDK4, β-Actin, histone H3, and secondary antibodies horseradish peroxidase-linked anti-mouse IgG, anti-rabbit IgG, and anti-goat IgG were purchased from Santa Cruz Biotechnology (Santa Cruz, CA). Antibodies for cyclin D1, cyclin D2, cyclin E, CDK6 and p53 were obtained from Cell Signaling Technology, Inc. (Denver, MA). MG132, Valproic acid (VPA) and other chemicals of analytical grade were purchased from Sigma-Aldrich Corp. (St. Louis, MO).

### Cell lines and cell culture conditions

The human melanoma cells lines A375, Hs294t, and SK-Mel28 were purchased from the American Type Culture Collection (Manassas, VA). All cell lines were cultured as monolayers in Dulbecco's Modified Eagle's Medium, while SK-Mel119 and SK-Mel28 in RPMI-1640 medium, and supplemented with 10% heat-inactivated fetal bovine serum (Hyclone, Logan, UT), 100 μg/ml penicillin and 100 μg/ml streptomycin and maintained in an incubator with 5% CO_2_ at 370C. Normal human epidermal melanocytes (NHM) were obtained from Cell Culture Core Facility of Skin Diseases Research Center at the University of Alabama at Birmingham, Birmingham, AL, and were cultured in HMGS supplemented melanocytes growth medium-254 (Life Technologies, Grand Island, NY).

### Cell viability and colony formation assays

The effect of GTPs or valproic acid on the viability of cells was determined using the 3-(4,5-dimethylthiazol-2-yl)-2,5-diphenyltetrazolium bromide (MTT, Sigma Chemical Co.) assay as described previously [10]. The color absorbance was recorded at 540 nm. The effect of GTPs or valproic acid on melanoma cell viability was determined relative to the cell viability of control cells (non-GTPs- or non-valproic acid-treated) that were assigned an arbitrary value of 100%. To assess the anti-colonogenic potential of GTPs on melanoma cell lines, 2000 cells were suspended (per well of six-well culture plates) in a complete medium. After 4 days of cell seeding, the cells were treated with GTPs (40 and 60μg/ml) and incubated for another few days. The cultures were maintained in an incubator for 2 weeks and then colonies were detected and counted under Olympus microscope after staining them with crystal violet and using the CellSens software (Center Valley, PA).

### HDAC activity assay

HDAC activity in melanoma cells was determined using the colorimetric HDAC Activity Assay Kit (Active Motif; Carlsbad, CA) following the manufacturer's protocol, as also described previously [10]. Briefly, this assay kit provides: a positive control (a HeLa nuclear extract), a deacetylated HDAC assay standard, and a control inhibitor (trichostatin A; TSA) as well as the colorimetric HDAC substrate. The absorbance was measured using a microplate reader at 405 nm, and the HDAC activity is reported in terms of percent of control.

### Histone acetyltransferase (HAT) activity assay

HAT activity in human melanoma cells was determined using the EpiQuik^TM^ HAT Activity Assay Kit (Epigentek Group Inc.) following the manufacturer's instructions, and as described previously [10]. This assay kit is designed for measurement of total HAT activity. The amount of the acetylated histone, which is directly proportional to HAT enzyme activity, can be colorimetrically quantified through an ELISA-like reaction. The color absorbance was recorded using a microplate reader at 450 nm, and the HAT activity is reported in terms of percent of control (non-GTPs-treated cells).

### Cell lysates and western blot analysis

Following treatment of different melanoma cells with GTPs or any other agent for the indicated time periods, the cells were harvested, washed with cold PBS and lysed with ice-cold lysis buffer supplemented with a cocktail of protease inhibitors, as detailed previously [15, 21]. Proteins were resolved using gel electrophoresis on 10-12% SDS-PAGE and transferred onto a nitrocellulose membrane. After blocking the non-specific binding sites, the membrane was incubated with the primary antibody at 40C overnight. The membrane was then incubated with the appropriate peroxidase-conjugated secondary antibody, and the specific protein bands were detected using the enhanced chemiluminescence reagents. Equal protein loading on the gel and on the membrane was verified by stripping the membrane and re-probing with an anti-β-actin antibody for cytoplasmic proteins and an anti-Histone H3 antibody was used for nuclear proteins.

### Comet assay for the analysis of DNA damage

GTPs-induced DNA damage in melanoma cells was determined using the Comet assay, as detailed previously [21, 38]. Briefly, cells were treated with GTPs (0, 20, 40, and 60 μg/ml) for 48 h in a complete medium; then cells were harvested and resuspended in ice-cold PBS buffer. Approximately 1x 10^4^ cells in a volume of 75 μl of 0.5% (w/v) low-melting-point agarose were pipetted onto a frosted glass slide coated with a thin layer of 1.0% (w/v) agarose, covered with a coverslip, and allowed to set on ice for 10 minutes. Following removal of the coverslip, the slides were immersed in ice-cold lysis solution. After 2 h at 4^0^C, the slides were placed into a horizontal electrophoresis tank filled with electrophoresis buffer and subjected to electrophoresis for 30 min at 300 mA. Slides were transferred to neutralization solution (0.4 M Tris-HCl; pH 7.5) for 5 min for washing and stained with ethidium bromide. DNA damage was detected and images were obtained under Olympus microscope equipped with Q-Color 5 camera with CellSens software. For each sample, the tail lengths (μM) of minimum of 6-10 cells were analyzed. The length of the Comet was quantified as the distance from the center of the cell nucleus to the tip of the tail and expressed as a mean ± SD.

### Statistical analysis

The statistical significance of differences between control and GTPs- or Valproic acid-treated groups was calculated by using GraphPad software (San Diego, CA). Quantitative data are shown as mean ± SD. In each case *P*<0.05 was considered statistically significant.
